# Functional profiling of microtumors to identify cancer associated fibroblast-derived drug targets

**DOI:** 10.18632/oncotarget.21915

**Published:** 2017-10-20

**Authors:** Shane R. Horman, Jeremy To, John Lamb, Jocelyn H. Zoll, Nicole Leonetti, Buu Tu, Rita Moran, Robbin Newlin, John R. Walker, Anthony P. Orth

**Affiliations:** ^1^ Functional Genomics, Genomics Institute of the Novartis Research Foundation (GNF), San Diego, CA, USA; ^2^ Informatics, Genomics Institute of the Novartis Research Foundation (GNF), San Diego, CA, USA; ^3^ Discovery Pharmacology, Genomics Institute of the Novartis Research Foundation (GNF), San Diego, CA, USA; ^4^ Genetics, Genomics Institute of the Novartis Research Foundation (GNF), San Diego, CA, USA; ^5^ Genomics Screening Core, Genomics Institute of the Novartis Research Foundation (GNF), San Diego, CA, USA; ^6^ Histology, Genomics Institute of the Novartis Research Foundation (GNF), San Diego, CA, USA; ^7^ Present address: Helix, San Diego, CA, USA

**Keywords:** spheroid, 3D, screening, GPR68, CAF

## Abstract

Recent advances in chemotherapeutics highlight the importance of molecularly-targeted perturbagens. Although these therapies typically address dysregulated cancer cell proteins, there are increasing therapeutic modalities that take into consideration cancer cell-extrinsic factors. Targeting components of tumor stroma such as vascular or immune cells has been shown to represent an efficacious approach in cancer treatment. Cancer-associated fibroblasts (CAFs) exemplify an important stromal component that can be exploited in targeted therapeutics, though their employment in drug discovery campaigns has been relatively minimal due to technical logistics in assaying for CAF-tumor interactions. Here we report a 3-dimensional multi-culture tumor:CAF spheroid phenotypic screening platform that can be applied to high-content drug discovery initiatives. Using a functional genomics approach we systematically profiled 1,024 candidate genes for CAF-intrinsic anti-spheroid activity; identifying several CAF genes important for development and maintenance of tumor:CAF co-culture spheroids. Along with previously reported genes such as WNT, we identify CAF-derived targets such as ARAF and COL3A1 upon which the tumor compartment depends for spheroid development. Specifically, we highlight the G-protein-coupled receptor OGR1 as a unique CAF-specific protein that may represent an attractive drug target for treating colorectal cancer. *In vivo*, murine colon tumor implants in OGR1 knockout mice displayed delayed tumor growth compared to tumors implanted in wild type littermate controls. These findings demonstrate a robust microphysiological screening approach for identifying new CAF targets that may be applied to drug discovery efforts.

## INTRODUCTION

Growing recognition that microenvironments may contribute to cancer development and progression has led to increased interest in understanding the tumor:niche relationship and in mining the niche for therapeutically tractable targets. Subsequently, a plethora of research investigating stromal targets for use in chemotherapy has surfaced over the last few years, supporting a more nuanced view of the contribution of the stromal niche to neoplastic transformation (reviewed in ref [1]).

The tumor milieu is a complex and heterogeneous assembly of different types of mesenchyme- and hematopoietic-derived stromal cells, which vary between tissue and tumor types [2]. Although transformed, cancer-causing cells initiate tumorigenesis, accessory stromal cells are recruited early on in tumor development [3]. Far from being unwitting accomplices in tumor progression, stromal cells directly contribute to the growth and maintenance of the tumor [4] and play large roles in therapy resistance [5]. Specifically, cancer-associated fibroblasts often constitute the bulk of the tumor mass and enhance tumor growth through secretion of protumorigenic cytokines and growth factors as well as upregulating ligand networks that stimulate receptors on cancer cells [6, 7]. Further, CAF components contain independent prognostic information where increased CAF populations have been shown to be associated with poor prognoses and overall reduced patient survival [8, 9]. However, with the exception of several CAF candidates that are ubiquitously found in many different tumor types (namely *WNT*, *HGF* and *FAP*) [10], the fibroblast component of tumors has yet to be thoroughly or selectively profiled for viable drug targets.

One potential reason for the lack of validated CAF targets is the difficulty in mimicking tumor:CAF interactions *in vitro*. Although conventional 2D approaches have proven effective at interrogating the intracellular origins of tumorigenesis, they lack the higher-order tissue structuring associated with *in vivo* cellular biology; and in that respect 2-dimensional (2D) cellular systems are poor surrogates for patient tumors [11]. Consequently, 2D oncology studies can provide misleading data and are poorly predictive of *in vivo* responses [12, 13]. Culturing cells in 3-dimensional (3D) space addresses relevant tumor architecture and, in particular, the multicellular tumor spheroid model is increasingly recognized as a useful technology for mimicking tumor biology [14]. Cells grown as 3D spheroids have been shown to be more “organotypic” of patient tumors in the context of cell-to-cell and cell-to-matrix interactions, cytoskeletal arrangement, cell receptor distribution and genetic and biochemical signaling networks [14, 15]. The importance of the spheroid model for oncology research is revealed by the increasing use of this platform for phenotypic drug discovery [16–19].

One particular aspect of the tumor microenvironment that is mirrored in spheroids is peritumoral acidosis. The interior pH of solid tumors is acidic due to increased fermentative metabolism and poor oxygen perfusion [20]. This acidic microenvironment promotes degradation of the extracellular matrix (ECM) by metalloproteinases [21] and increases angiogenesis through the release of VEGF [22]. The *in vivo* acidic microenvironment is replicated in the core of multicellular tumor spheroids [23], enabling the study of pH-reactive genes and proteins and their involvement in tumor growth. A protein important for pH-dependent tumor biology and sensitive to 3D structural growth is the ovarian cancer G-protein-coupled-receptor-1 (*OGR1*, also called *GPR68*). *OGR1* is a pH-sensing GPCR that is involved in maintaining pH homeostasis, functioning as a proton-sensing receptor that stimulates inositol phosphate formation at pH < 6.8 [24]. Intriguingly, transgenic *Ogr1*^−/−^ mice exhibit a profound reduction in sensitivity to tumor formation [25]. Allograft transplantation of transgenic adenocarcinoma mouse prostate (TRAMP) cancer cells lead to significant tumor restriction in *Ogr1*^−/−^ mice [26], indicating *Ogr1* appears to have protumorigenic properties when expressed in the murine tumor host environment. In humans, *OGR1* is expressed in certain subsets of immune cells including macrophages, neutrophils, CD4^+^ or CD8^+^ T-cells, [26] and in vascular smooth muscle cells [27]. Recent efforts to drug OGR1 identified several allosteric modulators of OGR1 function, yet the protein remains “pharmacological dark matter” [28].

In the present study we sought to identify CAF-derived factors that are important for colorectal cancer (CRC) tumor formation. By taking a reductionist approach to cancer:CAF interactions, we employed co-culture spheroids in a phenotypic screening platform composed of human CRC cells and normal human colon fibroblasts. We refer to these structures as microtumors and used them in an arrayed-well high-content genomics screen to dissect CAF:cancer paracrine signaling. We identify several novel CAF proteins important for human CRC tumor formation. In particular, OGR1 is characterized as a CAF-specific protein important for 3D tumor structural growth both *in vitro* and *in vivo*. This is the first report of spheroids used in high-throughput genomics-based phenotypic profiling and represents a first-in-class assay platform to interrogate CAF:cancer interactions.

## RESULTS

### Design of the co-culture microtumor screening platform

**Figure 1:**
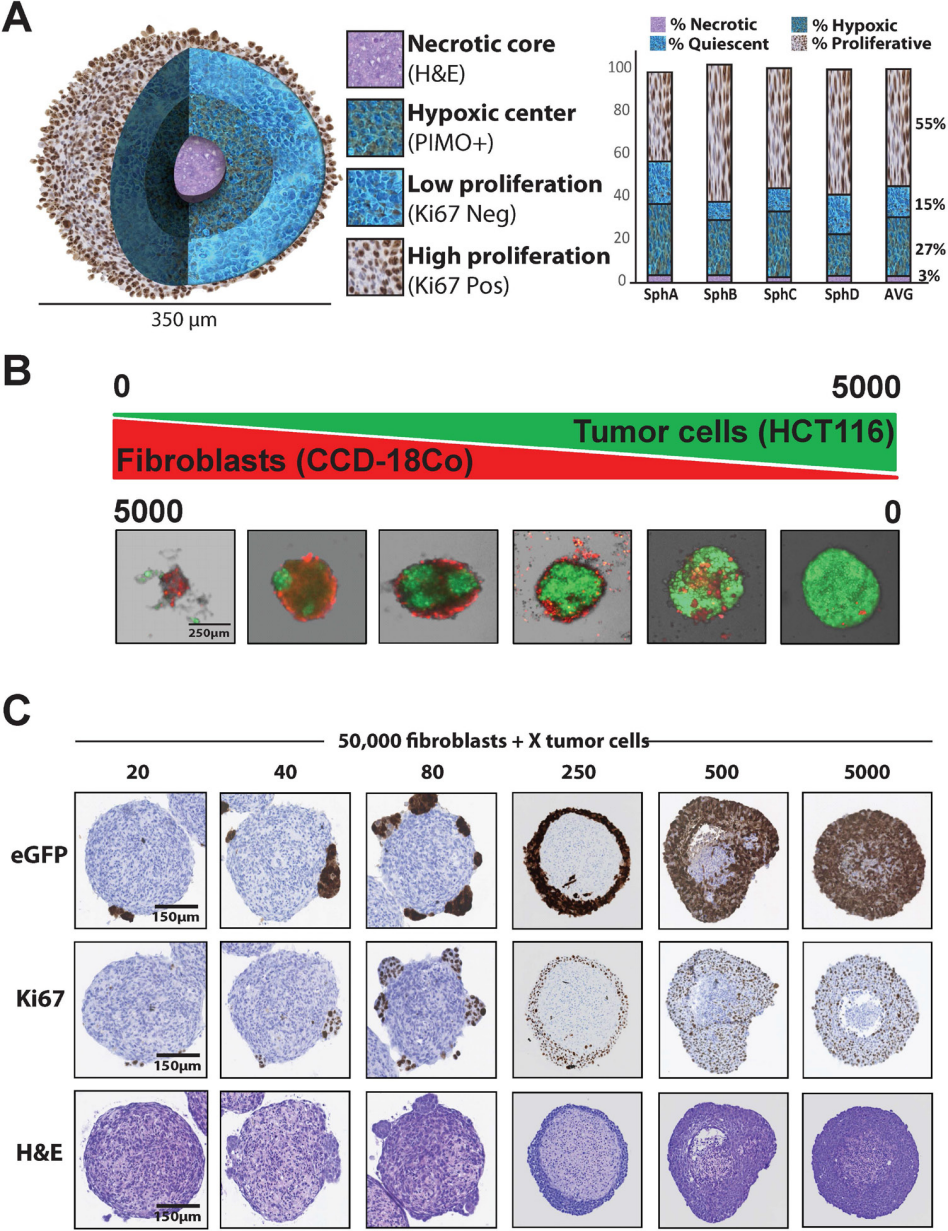
Analysis of tumor:fibroblast spheroid biology (**A**) Composite of immunohistochemistry images showing important spheroid regions. The outer shell of the spheroid contains highly proliferating cells (Ki67 high), which surround a relatively quiescent zone (Ki67 low). The hypoxic center, characterized by pimonidazole adducts (PIMO+), mantels a necrotic core (H&E), characterized by lacunae of necrosis. Bar graph shows relative proportions of 4 different spheroids, all 350 µm in diameter. (**B**) CCD-18Co normal human colon fibroblasts (stained in red) do not form 3-dimensional spheroid structures alone. Titering in HCT116 human colon carcinoma cells (expressing eGFP) to CCD-18Co fibroblasts causes their polarization and formation of 3D spheroids. (**C**) Histology sections of tumor:stroma co-culture spheroids. 50,000 CCD-18Co normal human colon fibroblasts were mixed with increasing numbers of HCT116-eGFP cancer cells, fixed, sectioned and stained for cancer cell orientation (eGFP) and proliferation (Ki67).

With the goal of creating a more bio-mimetic tumor screening assay for high content drug discovery, we mixed colon tumor cells with normal colon fibroblasts in a 3-dimensional microtumor (spheroid) that could be cultured and assayed in 384-well microtiter plates. The purpose of using normal colon fibroblasts and not primary CAFs derived from a patient tumor was because we were interested in studying the conversion of normal mesenchyme-derived cells to activated CAFs and how those CAF-specific genes affect tumor development in a paracrine manner. Morphologies of the microtumor spheroids were characterized similarly to spheroids of larger dimensions in that they contained proliferative outer shells (Figure 1A, Ki67 Pos and Supplementary Figure 1A, Live Tracker) which surround an area of low proliferation (Figure 1A, Ki67 Neg). The center of the microtumor spheroid displays hypoxia (pimonidazole^+^ hypoxic adducts) due to low oxygen diffusion into this space (Figure 1A, PIMO+) and the core of the spheroid is necrotic and characterized by low pH and lacunae of necrosis (Figure 1A, H&E and Supplementary Figure 1A, pH Tracker, Lyso Tracker) [14, 29]. These various hierarchical regions are arranged in proportions similar to patient tumors in that large portions of the cells are actively proliferating, even though there may be significant areas of hypoxia (Figure 1A, bar graph).

During the development of the co-culture colon tumor:stroma spheroid model we observed an interesting phenomenon concerning untransformed colonic fibroblasts. The normal human colon fibroblast cell line, CCD-18Co, does not form 3D structures *in vitro*, only loose cellular aggregates, but they can be polarized into distinct 3D structures through the addition of colorectal cancer cells to the culture. Cell titering assays using HCT116 human colorectal carcinoma cells engineered to express the enhanced green fluorescent protein (HCT116-eGFP) revealed that only 5 HCT116-eGFP cells in 5,000 CCD-18Co normal colon fibroblast cells (stained red) were required to form a 3D spheroid (Figure 1B). The tumor cells appear to serve as an anchoring point for the fibroblasts and result in their distinct 3D adhesion (Figure 1C, left panels). Increasing the ratio of colon tumor cells to fibroblast cells in these spheroid assays resulted in the tumor cells encompassing and invading the fibroblasts, akin to the mechanism of tumor development *in vivo* (Figure 1C, right panels). Intriguingly, we were able to show that factors secreted from the HCT116 tumor cells were responsible for this fibroblast polarization. By using conditioned media (CM) from HCT116 cells grown either in a 2D monolayer or as 3D spheroids, we demonstrated that secreted proteins within the HCT116 CM were able to induce spheroid formation of CCD-18Co fibroblast cells (Supplementary Figure 1B).

### High content functional genomics screen of microtumors identifies protumorigenic CAF genes

In order to dissect the biology of how normal colon fibroblasts are hijacked by associated tumor cells into forming 3D spheroid structures, we performed a high-content, arrayed-well functional genomics screen aimed at identifying candidate fibroblast-specific factors necessary for the formation of co-culture microtumors.

**Figure 2:**
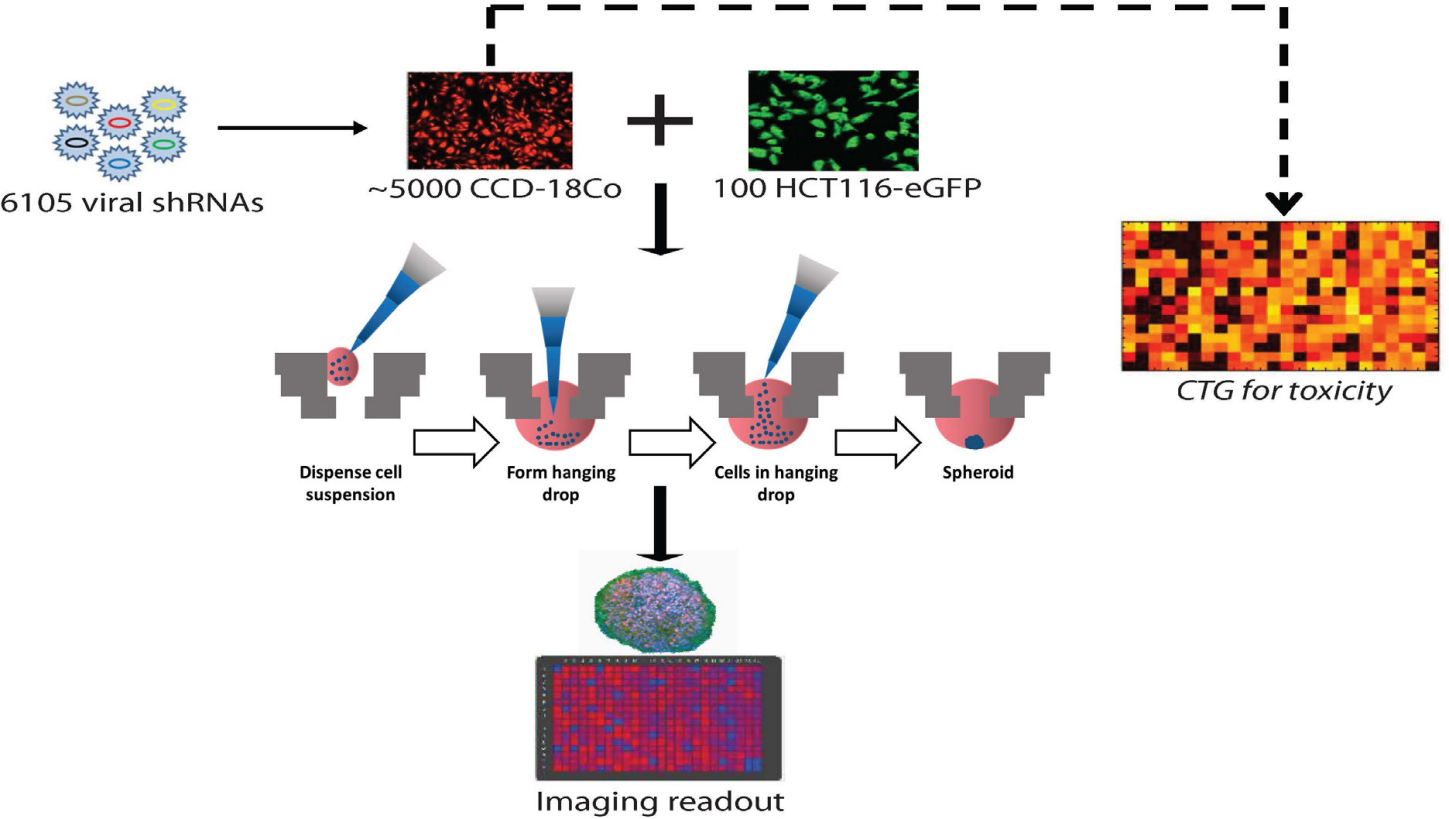
Schematic of high-content genomics screen to identify stromal-specific factors necessary for tumor spheroid formation A custom genomics library of approximately 6,105 shRNAs, corresponding to 1,024 genes, was used to infect CCD-18Co normal human colon fibroblasts. After expansion of puro-selected CCD-18Co transductants, a small aliquot of CCD-18Co cells was assayed for viability in a CellTiter-Glo (CTG) assay. Approximately 5,000 CCD-18Co fibroblast cells (expressing one target shRNA) were then stained red and mixed with 100 HCT116-eGFP colon cancer cells in HTS spheroids. After 4 days, spheroids were assayed by fluorescence cytometry and spheroid formation was calculated.

Using a variety of genomics data inputs such as primary colorectal cancer gene expression signatures from The Cancer Genome Atlas Network (TCGA) we nominated 1,024 genes for shRNA-mediated depletion in the fibroblast component of the fibroblast:cancer 3D model system (Supplementary Table 1). We then assembled 6,105 unique lentiviral shRNAs targeting these genes (5-6 shRNAs per gene) and systematically depleted them in the CAF compartment of co-culture CAF:cancer spheroids in arrayed wells. After 72 hours, spheroid formation was quantitated using a laser-scanning fluorescence cytometer (Figure 2).

**Figure 3:**
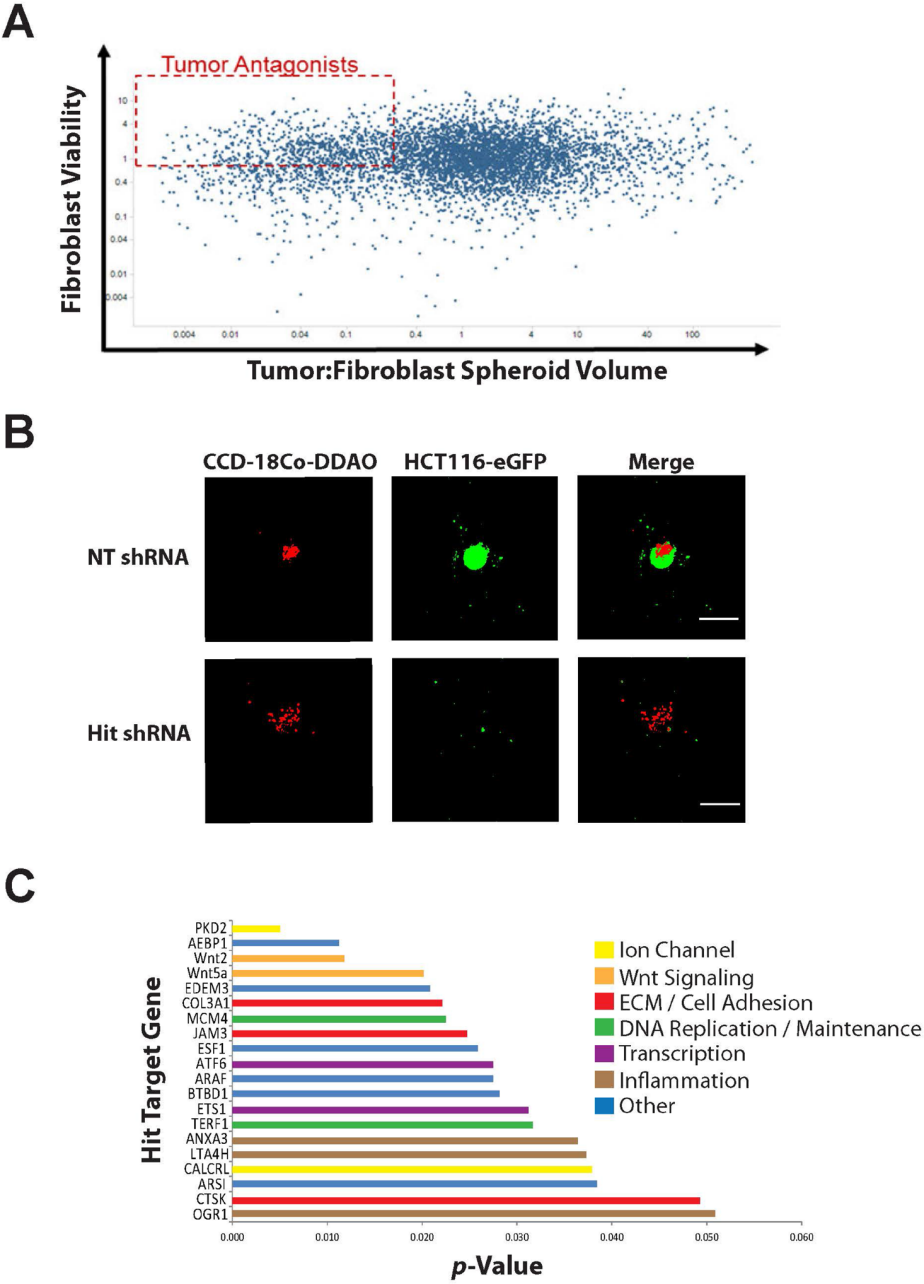
Results of high-content spheroid genomics screen (**A**) Dot plot of results from screen of 6,105 shRNAs plotting eGFP spherical volume (tumor:fibroblast spheroid volume) by CCD-18Co area (fibroblast viability). Tumor antagonist “hits” were characterized as those shRNAs that reduced spheroid size by more than 60%, but had no more than a 30% effect on fibroblast viability (dotted red box). (**B**) Acumen images show tumor:stroma spheroids from a non-targeting (NT) control shRNA and a hit shRNA (tumor antagonist). (**C**) *P*-values for top 20 hits from the screen. All targets had multiple shRNAs scoring as hits, mitigating the likelihood of off-target effects. Scale bar indicates 500 µm.

The 3D tumor:stroma spheroid screen yielded a broad range of phenotypes. Genes classified as hits from the primary screen were defined as those reducing microtumor formation by more than 60%, but reducing 2D CCD-18Co viability less than 30% (Figure 3A, 3B and Supplementary Figure 2). After reconfirmation assays, we determined 39 genes scored as positive hits from the screen and 20 of those hits demonstrated *p* values less than 0.05. Strikingly, these top 20 hit genes were enriched for *WNT* signaling, extracellular matrix remodeling, cell adhesion and inflammation (Figure 3C).

### Fibroblasts from co-culture tumor spheroids display gene signatures similar to patient CAFs

**Figure 4:**
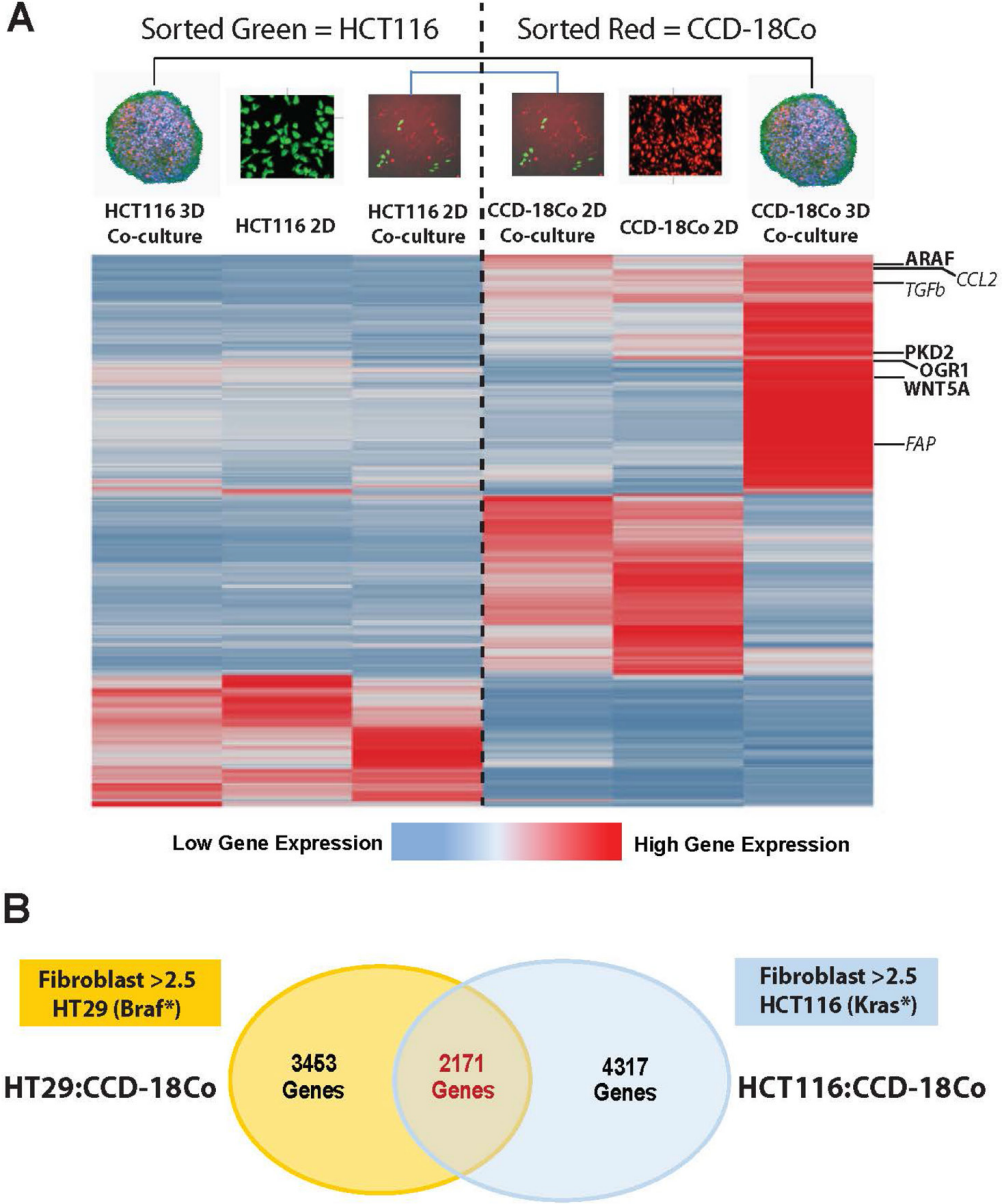
CAF signatures are unique to 3D co-culture tumor spheroids and stromal hit genes are conserved over numerous tumor types (**A**) Heat map of RNAseq data from purified populations of tumor:stroma cultures: HCT116-eGFP cells (grown in 2D monoculture, 2D co-culture or 3D co-culture spheroids) and CCD-18Co cells (grown in 2D monoculture, 2D co-culture or 3D co-culture spheroids). The battery of genes activated in CCD-18Co cells upon 3D co-culture (right most column) contains CAF signature genes (in italics) as well as many of the hit genes from the genomics screen (in bold). (**B**) The same RNAseq experiment was performed with HT-29 colorectal carcinoma cells and CCD-18Co cells and the Venn diagram shows CCD-18Co CAF signature genes (> 2.5 fold increase in 3D/2D) conserved between the two experiments.

To triage hit genes from the screen, we subjected sorted spheroid cell populations to gene expression analyses to determine which hit genes were upregulated in CCD-18Co cells upon 3D co-culture microtumor growth and, therefore, likely involved in distinct tumor-related biology. HCT116-eGFP cells and CCD-18Co cells (labeled with red tracking dye) were grown either separately in 2D monoculture, together in 2D co-culture, or together in 3D co-culture tumor spheroids, sorted by fluorescence-activated cell-sorting (FACS) and subjected to RNAseq. We observed a unique cluster of genes upregulated in CCD-18Co cells only when grown with colorectal tumor cells in 3D spheroids (Figure 4A). Co-culture of the fibroblast cells with tumor cells in 2D did not produce this distinct gene signature, indicating 3D microphysiological growth was required for gene activation. Within the fibroblast activated gene cluster were many canonical CAF-signature genes associated with an activated or polarized fibroblast cell population (*TGF*β [30], *CCL2* [31], *FAP* [32], Figure 4A, italicized genes). Interestingly, a number of hit genes from the genomics screen were also found to be upregulated in CCD-18Co fibroblasts only in the 3D co-culture tumor spheroid (e.g. *ARAF*, *PKD2*, *OGR1*, *WNT5A*; Figure 4A bold genes, Supplementary Figure 3 and Supplementary Table 2) and these lead targets were advanced into further discovery efforts (Supplementary Figure 2).

HCT116 colorectal carcinoma cells harbor the mutated *KRAS*G13D oncogene (common to 35-45% of CRC patients [33]) and we sought to ascertain whether the cancer cell-induced alteration of CCD-18Co fibroblast gene expression was exclusively KRAS-dependent. To assess this we repeated the gene expression experiments, substituting *BRAF*V600E-driven (common to approximately 15% of CRC patients [34]) HT-29 CRC cells for the KRAS-driven HCT116 cells. HT-29-eGFP tumor cells and CCD-18Co cells were cultured alone or together as before and subjected to RNAseq. We observed a similar phenomenon where CAF-like gene signatures in fibroblasts arose only when grown with HT-29 tumor cells in 3D co-culture spheroids.

Intriguingly, 63% of the genes activated in fibroblasts during microtumor growth with HCT116 KRAS-driven tumor cells were also activated when cultured with HT-29 BRAF-driven tumor cells (Figure 4B and Supplementary Table 3). The substantial degree of conservation in 3D tumor-induced CAF gene expression suggests that activated genes are directly responsive to microtumor structural growth largely independent of the molecular driver of the tumor cells.

**Figure 5:**
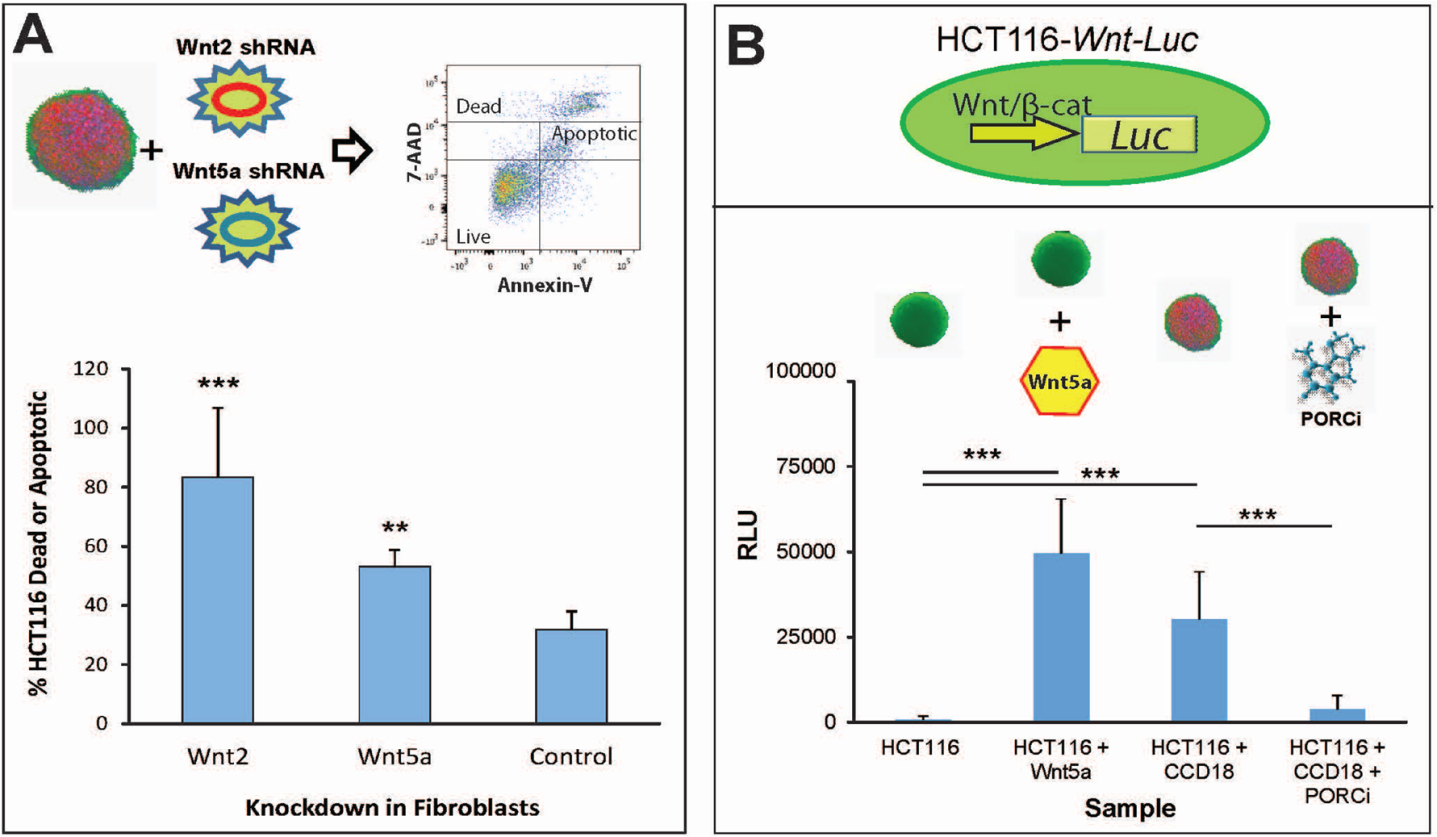
CAF-secreted WNT feeds tumor cell growth and spheroid formation (**A**) Apoptosis analysis of HCT116 cells grown in co-culture spheroids with CCD-18Co cells harboring either *WNT2* or *WNT5A* shRNA-mediated depletion. (**B**) Luciferase signal of HCT116 cells expressing a WNT/β-catenin luciferase reporter grown as monoculture spheroids, monoculture spheroids + purified exogenous Wnt5a protein, co-culture spheroids or co-culture spheroids + porcupine inhibitor GNF-1331(PORCNi). ^**^p < 0.01, ^***^p < 0.001.

One family of genes whose expression in normal fibroblasts significantly increases during 3D co-culture with tumor cells is the WNT family (7/19 *WNT* genes increased > 2.5-fold). To functionally confirm paracrine signaling between the colon fibroblasts and colon tumor cells in co-culture spheroids, we used the WNT signaling pathway as a benchmark example of crosstalk modeling. We used virally-encoded shRNAs targeting either *WNT2* or *WNT5A* to deplete these genes in CAF cells and then mixed CAF cells with HCT116-eGFP cells to create WNT-negative tumor:CAF spheroids. Apoptosis analysis of tumor:CAF spheroids using Annexin-V/7-AAD staining revealed large portions of apoptotic cells within the control samples, indicating that under basal conditions the necrotic core and hypoxic areas within a tumor spheroid can harbor many apoptotic cells. However, large increases in apoptosis were observed when depleting *WNT* paracrine signaling within the microtumors; 50% of HCT116 spheroid cells were dead or apoptotic when deprived of CAF-derived *WNT5A* and nearly 100% of HCT116 spheroid cells were dead or apoptotic when deprived of CAF-derived *WNT2* (Figure 5A). To further establish the importance of paracrine WNT signaling in this model we created a WNT/β-catenin-responsive HCT116 luciferase reporter line. HCT116-Wnt-Luc cells were grown either as monoculture spheroids or co-culture spheroids with CCD-18Co CAFs. Autocrine WNT signaling was undetectable in monoculture HCT116-Wnt-Luc spheroids whereas addition of exogenous recombinant hWNT5A protein to these spheroid cultures activated reporter activity (Figure 5B). Adding CCD-18Co fibroblast cells into the HCT116-WNT-Luc spheroids produced strong WNT-mediated luciferase reporter activity, that could be quenched by treating co-culture spheroids with a Porcupine inhibitor (GNF-1331) that blocks WNT secretion [35] (Figure 5B). These studies experimentally prove tumor cells are directly responsive to CAF-secreted factors in our co-culture tumor:CAF spheroid model.

### Depletion of stromal OGR1 reduces microtumor formation

**Figure 6:**
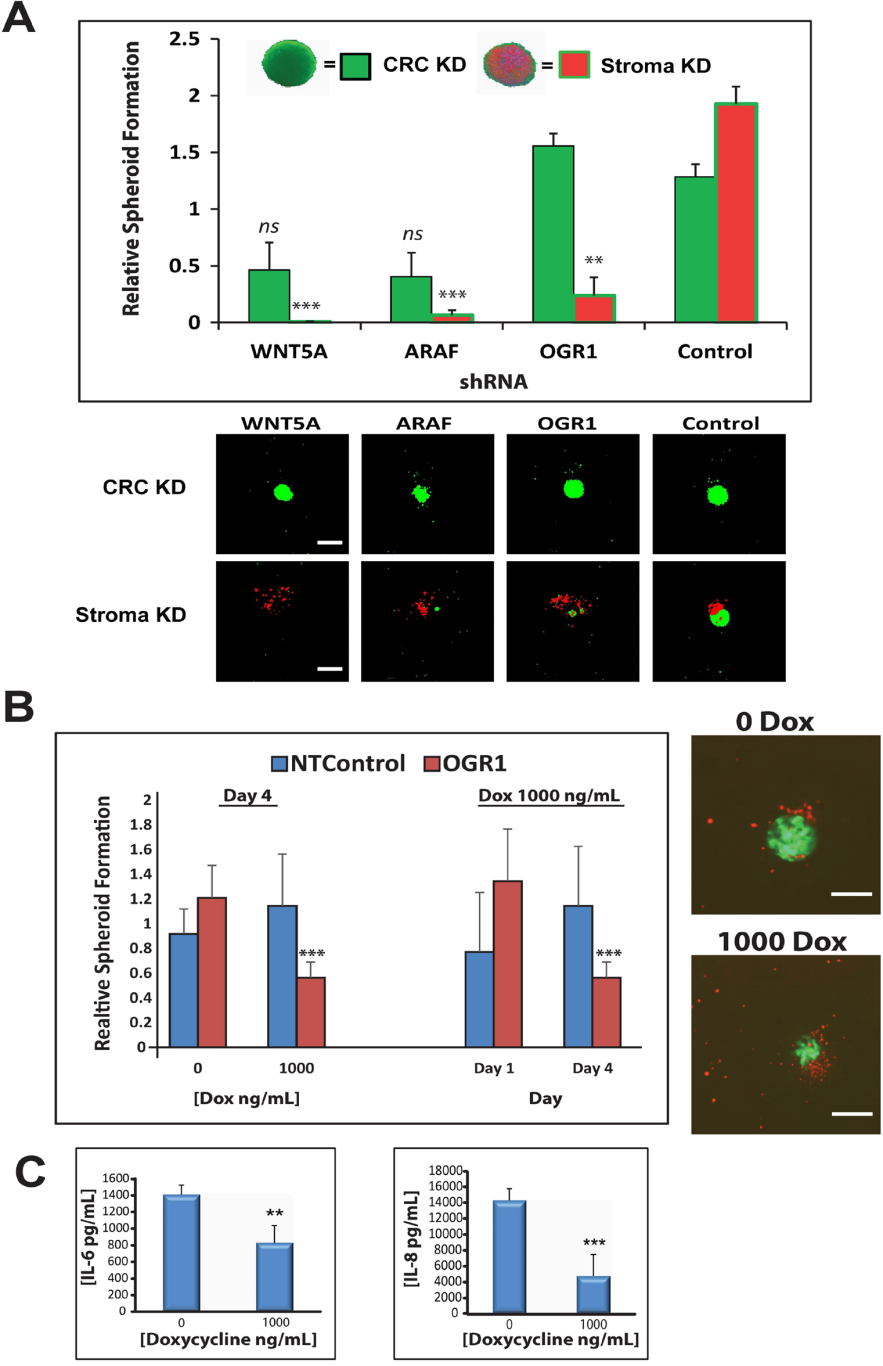
Prophylactic or therapeutic depletion of stromal OGR1 reduces tumor spheroid formation (**A**) Spheroid formation of either HCT116 monoculture spheroids (green bars) or HCT116:CCD-18Co co-culture spheroids (red/green bars) harboring constitutive shRNA-mediated knockdown of hit genes *WNT5A, ARAF, OGR1* or a non-targeting shRNA control. In the monoculture spheroids (green bars) the genes were knocked down in the HCT116 cells, in the co-culture spheroids (red/green bars) the genes were knocked down in the fibroblast cells. Images show representative spheroids from each sample. Error bars represent standard error of the mean. (**B**) Doxycycline-inducible depletion of *OGR1* in co-culture spheroids results in a therapeutic reduction of tumor spheroid size without concomitant fibroblast toxicity. Inset images are representative of spheroid biology +/− inducible *OGR1* depletion. (**C**) ELISA measurements of IL-6 and IL-8 in media from co-culture spheroids +/− inducible *OGR1* depletion. ^**^p < 0.01, ^***^p < 0.001, *ns* = not significant. Scale bars indicate 400 µm.

Many of the protumorigenic stromal genes identified from our screen and triaged through the RNAseq data were not detectably expressed in HT-29 or HCT116 CRC cells. In order to assess the CAF-intrinsic nature of our lead targets we reversed the 3D spheroid model, depleting hit genes with shRNAs in tumor cells instead of fibroblasts. Several target genes, including *ARAF* and *WNT5A*, demonstrated modest phenotypes when knocked down in the monoculture tumor spheroids (Figure 6A, green bars), but most shRNAs produced no discernable phenotype when depleted in the cancer cells (Supplementary Figure 4A) in contrast to their profound effects when depleted in the fibroblast component (Figure 6A, red bars and Supplementary Figure 4A). Further, these observations were not unique to HCT116 spheroids as we also showed that knockdown of key target genes within HT-29 CRC spheroids yielded no phenotype (Supplementary Figure 4B, green bars); but knockdown within the stromal cells of co-culture spheroids had a significant impact on spheroid formation (Supplementary Figure 4B, red bars). These data further support the hypothesis that the targets are mediating their actions in a purely stromal-driven manner, where genetic depletion of these genes in the tumor cells had little to no effect.

OGR1 is a proton-sensing GPCR found to be highly expressed in the CCD-18Co fibroblast population of our co-culture microtumors (Supplementary Table 3). Constitutive knockdown of *OGR1* in CCD-18Co fibroblasts using virally-delivered shRNAs significantly reduced microtumor spheroid size (Figure 6A). In order to ascertain if *OGR1* is a therapeutically-tractable CAF target, we engineered doxycycline-inducible shRNAs against *OGR1* and a non-targeting control shRNA (Supplementary Figure 4C). Inducible depletion of *OGR1* in the fibroblasts of pre-formed tumor:stroma spheroids significantly reduced spheroid size compared to the uninduced shRNA control spheroids (Figure 6B, inset images), suggesting a role for stromal *OGR1* in both microtumor formation and microtumor maintenance. Genetic depletion of *OGR1* has been associated with a reduction in the secretion of protumorigenic cytokines IL-6 and IL-8 [36, 37]. Consistent with these reports we detected significant reductions in secreted IL-6 and IL-8 in the media of CAF:tumor spheroids in which OGR1 was inducibly depleted (Figure 6C). These data reveal a possible mechanism of action for targeting stromal *OGR1*, which is a reduction in CAF-secreted proinflammatory cytokine signaling.

### *OGR1* is expressed in stromal compartments of patient tumors

**Figure 7:**
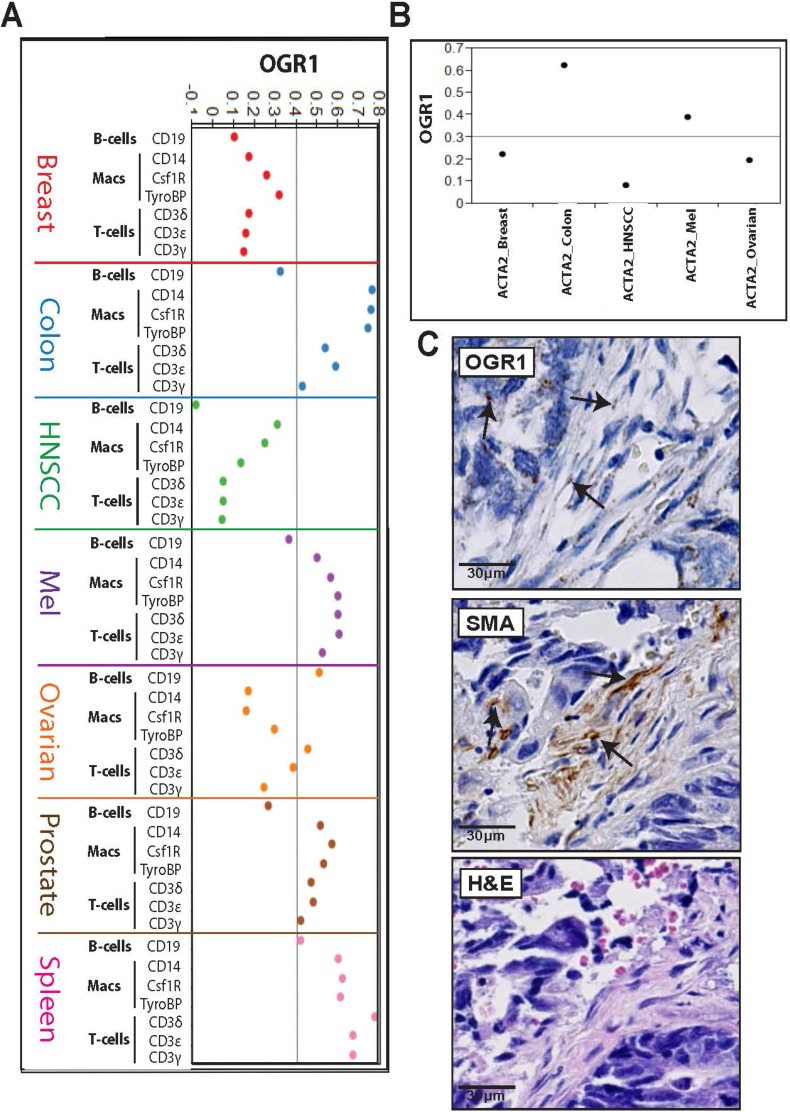
*OGR1* is expressed in hematopoietic and mesenchyme cellular compartments of colorectal cancer patient tumors (**A**) Pearson correlation coefficients of *OGR1* expression correlated to immune cell calibrator genes (*CD19* for B-cells; *CD14, CSF1R, TYROBP* for myeloid cells; *CD3*γ, δ and *ε* for T-cells) curated from TCGA data taken from patient tumors; score > 0.4 is significant (*p* < 0.001). (**B**) Pearson correlation coefficients of *OGR1* expression correlated to fibroblast cell calibrator gene smooth muscle actin (*ACTA2*) curated from TCGA data taken from patient tumors; score > 0.3 is significant (*p* < 0.001). (**C**) ISH/IHC of *OGR1* in human CRC tumor microarrays (TMAs). Dark spots marked with arrows indicate *OGR1* transcripts (for a summary of CRC TMA data see Supplementary Table 4) co-localized to smooth muscle actin (SMA) regions in human CRC TMA. Arrows indicate *OGR1* overlap with fibroblast cells and hematoxylin and eosin staining shows characteristic fibroblast cellular pathology at sites of *OGR1* expression. CRC: colorectal cancer, OvCA: ovarian cancer, HNSCC: head and neck squamous cell carcinoma, Mel: melanoma, BrCA: breast cancer, PRAD: prostate cancer. ISH: *in situ* hybridization, IHC: immunohistochemistry.

*OGR1* has been negatively linked to murine tumor formation [25, 26], and extending this paradigm to human tumor growth, we sought to detect *OGR1* expression within the stromal component of patient tumors. Gene expression profiles acquired from patient-derived primary tumors originate from numerous cell types. In some tumor types the amount of causal cancer cell mass may be negligible compared to non-cancerous stromal cell types found within a tumor, such as fibroblasts, vasculature or immune cells [38]. Bearing this in mind we looked through TCGA patient tumor RNAseq data for *OGR1* expression in B-cells, myeloid cells or T-cells of tumors excised from several different types of cancer patients. By employing immune cell calibrator genes (*CD19* for B-cells; *CD14, CSF1R* or *TYROBP* for myeloid cells; *CD3*γ*,* δ and ε for T-cells) we were able to discern the levels of *OGR1* expression within respective immune cell populations of patient tumors. Using this method we were able to detect significant expression of *OGR1* within the myeloid cells and T-cells of CRC patient tumors (Figure 7A, blue dots). Although *OGR1* expression most highly correlated to the myeloid cells of CRC patients, its expression also heavily correlated to myeloid cells found in melanoma tumors (Figure 7A, purple dots) and prostate tumors (Figure 7A, brown dots). The highest correlation of *OGR1* to *CD3*^+^ T-cells was likewise found in tumors from CRC, melanoma and prostate cancer patients. *OGR1* expression in B-cells was only detectable in ovarian tumors (Figure 7A, orange dots) and normal spleen (Figure 7A, pink dots), corroborating previously published data claiming *OGR1* is only found in macrophage and T-cell immune compartments of mouse tumors [26].

To clarify if *OGR1* expression correlates to the CAF compartments of human patient tumors we applied the same algorithms using the alpha smooth muscle actin (*ACTA2*) gene as a calibrator for CAFs. During our initial studies of tumor:CAF spheroids, we observed a significant upregulation of αSMA in the CCD-18Co fibroblasts during tumor cell co-culture and 3D growth, which led to its selection as a marker for representative CAF populations. Further, as RNAseq data from the tumor:CAF spheroids indicated paracrine TGFβ signaling, the use of αSMA as a CAF marker seemed appropriate given the established link between TGFβ signaling and αSMA expression [39]. Indeed, *OGR1* was highly correlated to *ACTA2* in tumors taken from CRC and melanoma patients, but not significantly correlated in breast, head and neck, or ovarian cancer patients (Figure 7B). These data reveal a previously unknown, CAF-intrinsic role for *OGR1* in human tumor formation.

Extending our informatics analysis of *OGR1* to archived tumor samples, we probed human CRC tumor microarrays (TMAs) for *OGR1* expression using *in situ* hybridization. Of the 95 colon adenocarcinoma samples present on the TMA, 26 yielded signal using the positive control probe (*PPIB*). Of those 26 samples, 11 tumors stained positive for *OGR1* expression and 2 of the 11 tumors scored very strongly for *OGR1* expression (Supplementary Table 4). Cells positive for *OGR1* expression also stained positive for αSMA, a marker of activated CAFs, and displayed typical fibroblast CAFs. pathologies by H & E staining (Figure 7C). These histology data confirm the informatics predictions that *OGR1* is found in the CAF compartments of CRC tumors.

### Ogr1-depleted TME affects early murine tumor growth

**Figure 8:**
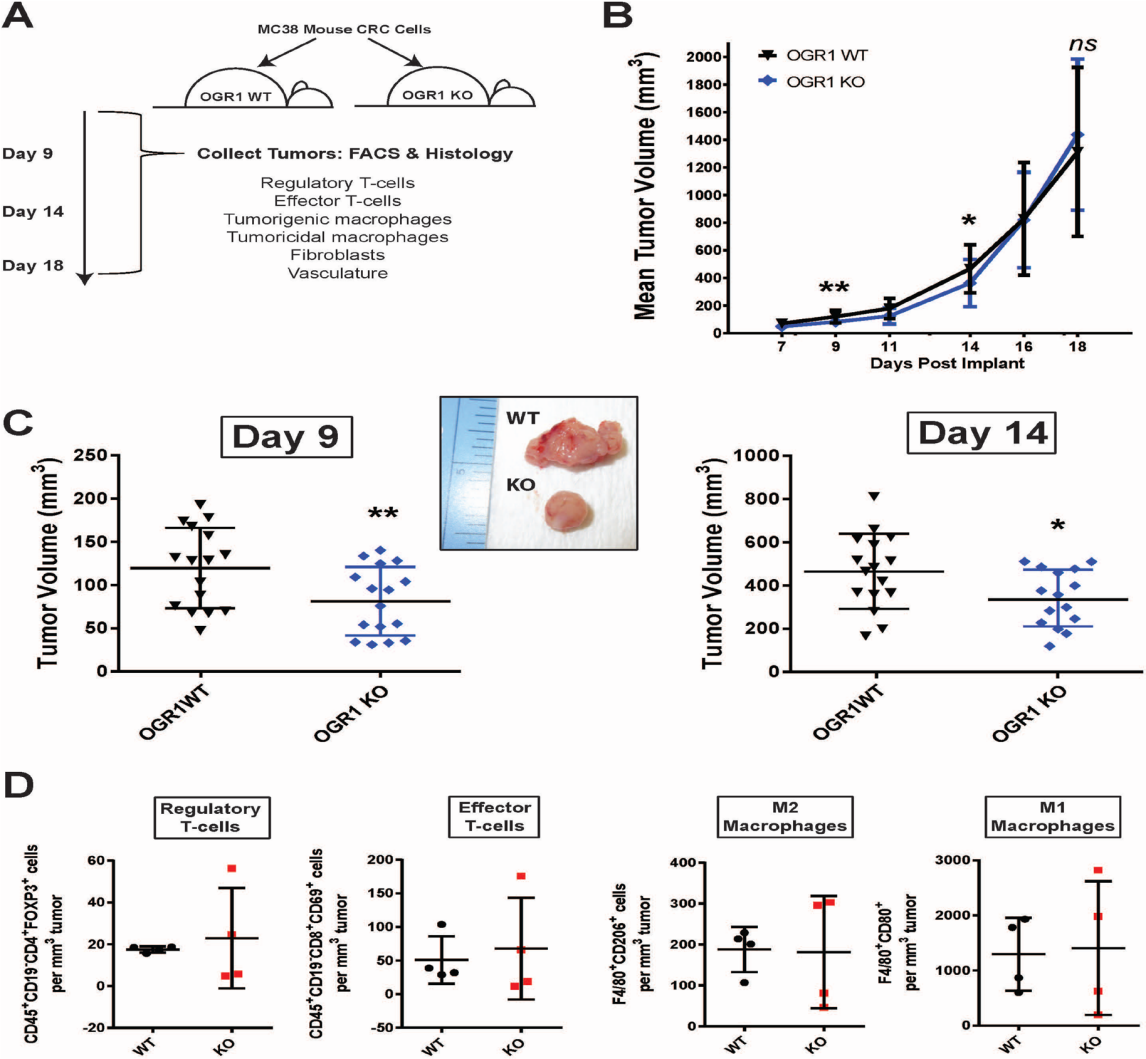
*Ogr1* knockout mice demonstrate stunted MC-38 tumor growth compared to wild type littermate controls (**A**) Schematic of tumor study workflow. Subcutaneous tumors were collected at day 9 and day 14 for FACS and histological analyses. (**B**) MC-38 tumor growth curves over 18 days in either *Ogr1* WT (black) or *Ogr1* KO (blue) murine littermates; mean tumor volumes calculated for 16 mice per group. (**C**) MC-38 tumor volumes calculated at day 9 and day 14 post-implant; inset image shows representative tumors at day 9. (**D**) FACS analyses of immune cell populations in day 14 tumors. Regulatory T-cells are characterized as CD45+CD19-CD4+FoxP3+, Effector T-cells are characterized as CD45+CD19-CD8+CD69+, M2 macrophages are characterized as F4/80+CD206+, and M1 macrophages are characterized as F4/80+CD80+. ^*^p < 0.05, ^**^p < 0.01, *ns* = not significant.

Ultimately, the most comprehensive method to analyze the effects of a TME-derived protein on tumor formation is *in vivo* tumor modeling. With this in mind we employed *Ogr1* transgenic mice in a tumor study using syngeneic MC-38 murine colorectal carcinoma cells. MC-38 cells were implanted subcutaneously into either *Ogr1* knockout mice or wild type littermates. Tumor formation was quantitated every 2-3 days for 18 days and tumors were harvested at either 400 mm^3^ for an early time point or 1400 mm^3^ for a late time point; at which time the tumors were subjected to histological and FACS-based analyses for differences in stromal cell populations (Figure 8A). Throughout the first 14 days of the tumor study, the *Ogr1* KO mice demonstrated significantly slower growing tumors compared to WT littermates. However, after 14 days the tumors in the KO mice exhibited similar growth kinetics to the WT mice, and the curves were indistinguishable by the end of the study (Figure 8B). It would appear that during the lag phase of tumor formation (up to day 11), the lack of *Ogr1* had a significant impact on tumor development (Figure 8C, Day 9). This impact lessened during the log phase of tumor growth (Figure 8C, Day 14) and after day 14 both WT and KO tumors exhibited identical growth curves. However, during early tumor development we noticed obvious pathological differences between tumors excised from KO and WT animals. Tumors from *Ogr1* KO animals were more fibrotic, less vascular and had more pronounced borders compared to tumors from *Ogr1* WT animals (Figure 8C, inset). We used multicolor flow cytometry to quantitate populations of regulatory T-cells, effector T-cells and macrophage subtypes within MC-38 tumors harvested at day 14. Although the tumors extracted from the *Ogr1* KO mice exhibited greater variability in these immune cell populations compared to the WT littermate controls, there were no significant differences observed in the quantities of any of these cell types (Figure 8D). Similarly, we used immunohistochemistry (IHC) to quantitate populations of T-cells (CD3), macrophages (Iba-1), vasculature (CD31), fibroblasts (SMA) and proliferative cancer cells (Ki67) in tumors excised from *Ogr1* KO mice or WT littermate controls and saw no significant differences in any of these populations by IHC (Supplementary Figure 5). It would seem that whatever stromal-derived factor was responsible for the retarded MC-38 tumor growth in *Ogr1* KO animals was not identified through our analytical methods. Certainly, these mechanisms warrant more granular investigations.

## DISCUSSION

The systematic study of CAF-derived paracrine signaling has heretofore been restricted by the paucity of *in vitro* cell models and the challenges confronting selective manipulation of the stromal component. Identification of protumorigenic CAF proteins in colorectal cancer requires co-culture of human colon cancer cells and relevant human colonic fibroblasts. Further, the two cell types must be assembled to achieve higher ordered structures that mimic *in vivo* tumor pathology. The 3D tumor:stroma spheroid model shown here replicates many aspects of CRC tumor biology observed in primary tumors, particularly paracrine WNT signaling. Both *WNT2* and *WNT5A* are upregulated 2-5 fold in the CAFs of both CCD-18Co:HCT116 and CCD-18Co:HT29 co-culture 3D spheroids (Supplementary Table 3), mimicking increases in stromal-derived WNT signaling observed in CRC patient tumors [40]. CAF-secreted WNT proteins were directly acting upon the associated CRC tumor cells (Figure 5), and these actions could be abrogated using WNT (Porcupine) inhibitors. Both *WNT2* and *WNT5A* were hits in the genomics screen, where knockdown of either *WNT* gene within the CAFs abolished microtumor spheroid formation. These data suggest that the phenotypic screening platform we developed to identify new CAF-derived protumorigenic factors is a robust *in vitro* surrogate for human CRC tumor formation.

In addition to *WNT* genes, several other previously-identified control genes also scored as hits in the microtumor screen. *COL3A1*, which encodes the pro-alpha1 chains of type III collagen, is found in connective tissue of most organs, has been repeatedly implicated in the development of tumor ECM, and has been shown to be produced by fibroblasts in response to TGFβ [41]. Similarly, *JAM3* (*JAM-C*), a junctional adhesion molecule, functions in endothelial cell adherence and has been shown to play imperative roles in melanoma lung metastases [42] and ovarian tumor development [43]; where genetic ablation of the target led to reduced metastasis and slowed tumor growth, respectively. *AEBP1* (*ACLP*), another ECM protein, is secreted by fibroblasts and myofibroblasts and has been shown to be involved in wound healing and tumor ECM remodeling [44, 45]. All three genes (*COL3A1*, *JAM3* and *AEBP1*) scored in the fibroblast-autonomous 3D CRC spheroid screen, reducing microtumor volume by 98%, 99.6% and 84%, respectively (Supplementary Table 1). Intriguingly, many of the genes implicated in ECM formation and remodeling that scored in the screen are responsive to TGFβ signaling. We observed significant upregulation of CAF-derived *TGFB3* in both the HCT116 (up 14-fold) and HT29 (up 8-fold) spheroid models, indicating a conserved mechanism that is agnostic to the genetic state of the tumor cells. Collectively, the scoring of these genes in our phenotypic screening assay validates and corroborates our experimental approach in modeling clinically-relevant 3D tumor biology.

One gene that was significantly upregulated in tumor spheroid CAFs (22-fold) and that scored as a hit in the microtumor screen was *OGR1*. This GPCR protein responds to hypoxia and subsequent acidosis through the binding of protons to its extracellular active site of histidine residues. Activation of *OGR1* has been shown to produce a protumorigenic response that includes activation and secretion of proinflammatory proteins such as COX2, IL-6 and IL-8 [36, 46]. IL-6 and IL-8 are multifunctional chemokines associated with reactive stroma [47] and which may play widespread and causative roles in cancer progression [48]. *IL6* and *IL8* genes were strongly upregulated in the CAF compartment of microtumors (approximately 200-fold for *IL6* and > 2000-fold for *IL8*) and secreted IL-6 and IL-8 proteins could be significantly repressed by knockdown of CAF *OGR1*. It is tempting to speculate that anoxia or hypoxia in spheroid cores leads to altered cellular metabolism and secretion of extracellular acidic metabolic byproducts. These acidic moieties then bind to and activate *OGR1* which in turn signals downstream, triggering the secretion of protumorigenic cytokines. *In vivo* IL-6 and IL-8 secretion would stimulate neoangiogenesis to deliver oxygen and nutrients to the growing tumor whereas *in vitro* the result could be the increased microtumor growth and maintenance we observed during our validation studies.

Translationally, we observed a significant correlation of *OGR1* expression to the T-cells, macrophages and fibroblasts of clinical CRC patient tumor samples, indicating potential functions of this protein in mesenchyme- and hematopoietic-derived components of the tumor microenvironment. Extrapolating bioinformatics data to wet bench scrutiny of CRC patient tumors, we were able to show by *in situ* hybridization the presence of *OGR1* mRNA within the CAF compartments of several CRC tumor types. This is the first report of *OGR1* localized to the CAF compartment of human tumors.

It has been reported that stromal *OGR1* contributes to prostate tumorigenesis in transgenic mouse models [26], and we sought to test those results using MC-38 syngeneic mouse CRC tumor implants. Although MC-38 cells are genetically distinct from HCT116 cells and human CRC tumors based on alleles harboring point mutations, they are a highly immunogenic tumor line syngeneic with the C57/Bl6 *Ogr1* mouse [49]. MC-38 tumor implants in *Ogr1* KO mice demonstrated significantly slower growth kinetics during early tumorigenesis compared to *Ogr1* WT littermate control animals. However, after 2 weeks the tumors in the *Ogr1* KO animals grew as fast as tumors in the *Ogr1* WT animals and by the termination of the study there were no differences in tumor size. It is conceivable that the hypoxia and acidic environment of the early avascular tumor implant results in the expression and activation of the OGR1 receptor. It is tempting to speculate that OGR1 signaling and subsequent release of IL-6 and IL-8 by OGR1-expressing cells may contribute to neoangiogenesis to feed the growing tumor and provide oxygen to perivascular tumor regions. This phenomenon has been observed with GPR4, another proton-sensing GPCR, where acidic activation of the receptor led to increased angiogenesis via IL-6, IL-8 and VEGF secretion [50]. As tumors become vascularized and oxygen penetration is reestablished, CAF-derived *OGR1* may shut off, which may explain similarities in tumor growth between WT and KO animals throughout the later stages of the study. Although the initial finding of the protumorigenic properties of *OGR1* was made using *in vitro* human CRC cell lines driven by clinically-relevant mutant proteins, we were able to demonstrate these pathologies translated to an *in vivo* mouse model of tumor growth. Given that the phenotype was not striking (particularly at the end of the tumor study) between *Ogr1* WT and *Ogr1* KO animals, we envision targeting the OGR1 protein as a combination therapeutic strategy where a standard of care chemotherapeutic would synergize with a stroma-targeting OGR1 antagonist. Certainly, these types of combination treatment modalities warrant more granular investigations.

Phenotypic screening in industrial drug discovery is an increasingly-recognized approach for the identification of new targets and compounds needed to combat cancer. However, targets and prospective drugs produced from phenotypic screening efforts are only as reliable as the disease models employed. The 3D co-culture tumor:stroma model presented here reveals interactions between cancer cells and fibroblasts that would not have been detected during conventional 2-dimensional cell culturing techniques. Further, the very nature of 3-dimensional microtumors more accurately mimics avascular tumor biology as well as cancer cell crosstalk in perivascular tumor regions. The conservation in CAF gene expression between *KRAS*- or *BRAF*-driven tumors indicates that any therapeutic targeting of CAF moieties may be efficacious for multiple types of CRC independent of the molecular driver, an important consideration when stratifying clinical patient populations. Exploiting CAF-derived proteins in conjunction with current standard of care may represent a new paradigm in treating solid tumors. By expanding the chemotherapeutic targeting space to include cancer cell-extrinsic factors we are exponentially increasing our odds at developing efficacious treatment approaches to alleviate the burden of disease.

## MATERIALS AND METHODS

### Creation of 384-well HTS co-culture spheroids

CCD-18Co normal human colon cells (ATCC) were cultured in EMEM + 10% FBS and stained with 1 µM CellTrace DDAO-SE (Life Technologies) according to the manufacturer’s instructions. CCD-18Co cells were then mixed in a 50:1 ratio (5000:100) with human colorectal carcinoma HCT116 cells (ATCC) expressing the enhanced green fluorescent protein (HCT116-eGFP) in EMEM + 10% FBS. All cell cultures tested negative for mycoplasma by Radil testing (IDEXX Bioresearch). Co-culture cell mixtures (5100 cells in 30µL medium) were then plated onto 384 well Perfecta Hanging Drop plates (3D Biomatrix) according to the manufacturer’s instructions. After 3 days spheroids were dropped into high throughput screening (HTS) 384 well imaging plates (Greiner) using the 384 well transfer tool (3D Biomatrix) for spheroid analysis.

### Imaging analysis of spheroids

Co-culture tumor:fibroblast spheroids dropped into 384 well imaging plates were analyzed for spheroid size (eGFP spherical volume) and fibroblast viability (DDAO total area) using the Acumen laser-scanning fluorescence cytometer (TTP Labtech). Briefly, HTS spheroid plates were read on the Acumen using the 488 nm and 640 nm lasers in conjunction with the appropriate filters and photomultiplier tubes (PMTs). Using the Acumen software, spherical volume (eGFP) as well as total area of CCD-18Co cell populations (DDAO) were quantitated. Spheroid imaging data was exported into Spotfire software (TIBCO) and analyzed by plotting spherical volume against CCD-18Co total area. Spherical volume measured the microtumor spheroid formation and CCD-18Co area was used as the readout for normal fibroblast toxicity.

### Spheroid functional genomics screening

A custom library of 6,105 shRNAs targeting 1,024 genes cloned into the pLKO lentiviral vector (TRC, Broad Institute) was selected and made into lentivirus in arrayed well format (1 shRNA per well) according to standard methods. Viral supernatants were stored in 96 well plates in a −80°C freezer.

CCD-18Co fibroblasts were plated in 96 well plates (Greiner) at 5000 cells/well in 50 µL EMEM + 10% FBS. The following day cells were infected with 50 µL of viral supernatants overnight. The following day puromycin was added (2 µg/mL) and positive transductants were selected for 72 hours. After 72 hours, EMEM without puromycin was added to expand positive transductants for 24 hours prior to the spheroid assay. After 24 hours of expansion CCD-18Co cells were trypsinized, washed and 1/5 of the well volume was plated into 96 well white-bottom plates (Greiner) that would later be used to measure CCD-18Co viability. The remaining 4/5 of the CCD-18Co culture (approximately 5000 cells) was stained and mixed with 100 HCT116-eGFP CRC cells in the spheroid formation assay as described above.

The 1/5 CCD-18Co cells plated onto 96 well white-bottom plates were incubated overnight and then subjected to the CellTiter-Glo (CTG) luminescent cell viability assay (Promega) according to the manufacturer’s instructions. The CTG assay was not used as it is characteristically to look for cytotoxic shRNAs but rather as a way to ensure that loss of spheroids was not merely due to low titer virus or cytotoxic shRNAs. Genes could only score if their targeting shRNAs yielded live fibroblasts after puromycin selection.

### Fibroblast signature genes and OGR1 relationship to ACTA2 and immune cell markers in human cancers

RNA-seq profiles of CCD-18Co cells in 2D mono-culture or in 3D co-culture with either HCT116 or HT29 were generated (see cell-sorting and RNA profiling sections). 3D fibroblast signatures were defined as genes up-regulated more than 2.5-fold in 3D vs 2D culture conditions for co-culture with either HCT116 or HT29. The fibroblast 3D signature was then assessed for association with fibroblasts in tumors in humans by assessing the correlation coefficients for each gene to the fibroblast marker smooth muscle actin (*ACTA2*). Spearman correlation coefficients for all genes versus *ACTA2* were derived from the TCGA colorectal adenocarcinoma data and the subset of genes in the 3D fibroblast signatures were assessed for enriched correlation to *ACTA2* in comparison to all other genes (Wilcoxon test). In a similar manner, the relationship of *OGR1* to markers of B (*CD19*), myeloid (*CD14, CSF1R, TYROBP*) and T cells (*CD3D, CD3E, CD3G*) was assessed by Spearman correlations using expression profiles from hundreds of human primary tumor biopsies from ovarian, CRC, HNSCC, melanoma (TCGA (https://tcga-data.nci.nih.gov/tcga/) and breast (PMID22522925) tissues.

### Inducible OGR1 depletion spheroid assays

CCD-18Co cells were infected with lentivirus expressing the pLKO-Tet-On-OGR1 doxycycline-inducible shRNA vector as described above. Cells were stained and mixed with HCT116-eGFP cells and put into hanging drops as described above. After 72 hours of spheroid formation, doxycycline (1000 ng/mL) was added to spheroid cultures for a further 96 hours. After 96 hours spheroid size was calculated by laser-scanning fluorescence cytometry as previously described and spheroids were imaged using confocal microscopy.

### *In vivo* tumor transplant study and flow cytometry of immune cell populations

All animal tumor studies were executed in compliance with IACUC guidelines and regulations under protocol P15-391. Approximately 250,000 MC-38 mouse CRC cells suspended in PBS were injected subcutaneously into the hip flanks of either *Ogr1* KO or *Ogr1* WT 8-10 week old female littermate mice (Deltagen). Sixteen mice per genotype were used for the tumor study to ensure statistical power. Tumor size was calculated using calipers every 2-3 days for 18 days. Groups were euthanized either at day 9, day 14 or when tumor sizes approached 2000 mm^3^ using IACUC-approved methods. Tumors were cut into two and half of the tumor was fixed for histological analyses and half the tumor was mechanically digested using a Covaris Cryoprep Pulverizer (Covaris) according to the manufacturer’s instructions. Cells were stained with the following FACS antibody combinations before being subjected to multicolor flow cytometry: Macrophage panel is anti-F4/80-FITC, anti-CD80-PE, anti-CD206-APC, anti-CD45-Pacific Blue (all from Biolegend) and DAPI (BD Biosciences); T-cell panel is anti-CD4-APC-H7 (BD Biosciences), anti-CD8-PE (BD Biosciences), anti-FoxP3-e660 (EBioscience), anti-CD69-FITC (BD Biosciences), anti-CD19-BV605 (Biolegend), anti-CD45-Pacific Blue (Biolegend) and DAPI (BD biosciences). Flow cytometry was performed on a FACSCalibur (BD Biosciences) and FACS data was analyzed using FlowJo V10 software.

### Histology of spheroids

Co-culture spheroids (prepared by HTS method described above) were fixed in 10% Neutral Buffered Formalin overnight and then placed in 70% EtOH. Spheroids were embedded in 3% NuSieve agarose using a camel hair fine paintbrush to ensure all spheroids were embedded within the same plane. The spheroid-agarose plug was then processed using a Thermo Shandon Tissue Processor using a shortened process protocol specifically for agarose embedded tissues, then embedded into a paraffin block. 5 micron-thick sections were then placed on positive charged slides using a serial step sectioning protocol in groups of 5 slides, with 3 sections per slide. The first slide was stained with Hematoxylin and Eosin, the second and third slides were immunohistochemically stained using markers to identify proliferative portions of the microtumor spheroid. All immunohistochemical stains were performed in an automated fashion using a Ventana Discovery XT except for pimonidazole. Anti-Ki67 (Thermo Scientific, rm-9106-s) was used at a 1:50 dilution and anti-eGFP (Lifespan, ls-c67081) was used at a 1:100 dilution. Pimonidazole staining (Hypoxyprobe) was performed according to the manufacturer’s instructions. Slides were scanned using a Hamamatsu Nanozoomer slide scanner at 20× magnification.

### Histological analysis of OGR1 in human tumors

Samples were fixed in 10% neutral-buffered formalin (NBF) for 24 h, followed by dehydration and infiltration with paraffin using an automated processor and then manual embedding in paraffin. Samples were sectioned at a thickness of 5 μm. Sample quality was assessed by performing RNAscope (Advanced Cell Diagnostics) analysis for mRNA of the housekeeping gene peptidylprolyl isomerase B (PPIB). The RNAscope assay is fully automated on the Roche Ventana Medical Systems DISCOVERY ULTRA platform. Sections were baked (60 min at 60°C) and deparaffinized on the instrument, followed by target retrieval and protease treatment. Probes were then hybridized for 2 h at 43°C followed by RNAscope amplification and DAB chromogenic detection using Ventana detection reagents.

### Histological analysis of MC-38 tumors excised from OGR1 transgenic mice

MC-38 mouse CRC tumors were excised from mice at either 400 mm^3^ or 1400 mm^3^ and fixed in 10% neutral-buffered formalin (NBF) for 24 h, then placed in 70% EtOH. Samples were embedded in paraffin and sectioned at a thickness of 5 μm. 5 micron-thick sections were then placed on positive charged slides using a serial step sectioning protocol in groups of 5 slides, with 3 sections per slide. The first slide was stained with Hematoxylin and Eosin and subsequent slides were immunohistochemically stained using markers to identify cancer cells (anti-Ki67), T-cells (anti-CD3), macrophages (anti-Iba-1), fibroblasts (anti-SMA) and vasculature (anti-CD31) (all from Abcam). All immunohistochemical stains were performed in an automated fashion using a Ventana Discovery XT. Slides were scanned using a Hamamatsu Nanozoomer slide scanner at 20× magnification.

### shRNA pool target selection

Previous pilot shRNA screening identified 5 genes affecting sphere formation of CCD-18Co fibroblasts and HCT116 colon cancer cells – Itgb1, Itgb5, Pdgfra, Pdgfrb and Vim. To enrich for additional candidate genes involved in 3D growth that were relevant to human colorectal cancer, TCGA colorectal adenocarcinoma expression data from 191 samples (https://tcga-data.nci.nih.gov/tcga, RSEM gene, downloaded on 9/5/2013) was used to identify genes correlated to the 5 seed genes. This resulted in identification of 1,744 unique genes (genes correlated per seed – *ITGB1 n* = 1,032, *ITGB5 n* = 25, *PDGFRA n* = 148, *PDGFRB n* = 434, *VIM n* = 600) with 423 genes correlated to two or more of the 5 seed genes. This set of genes was further filtered to those considered to be druggable, cell surface and/or secreted, resulting in the final screening set of 1,024 genes (see Supplementary Table 1).

### ELISAs

Conditioned media from wells containing co-culture spheroids were extracted and 10 µL was used to profile for proinflammatory cytokines using the Human Proinflammatory 7-Plex Tissue Culture Kit (Mesoscale Discoveries) according to the manufacturer’s instructions. ELISA plates were assayed on a Sector S 600 instrument and data were analyzed using MSD Discovery Workbench Software.

### RNAseq analysis

Total RNA isolation was done using Trizol and chloroform then followed by Qiagen RNeasy column purification. Ten nanograms of total RNA was used to make amplified cDNA following the Nugen Ovation RNA-Seq System V2. Twenty nanograms of cDNA was used to construct Illumina RNA sequencing libraries. Libraries were sequenced using 50 bp single reads on an Illumina HiSeq 1000. Reads were aligned to the human transcriptome using BWA [51]. Around 40 million reads per sample mapped to the human transcriptome. Expression values were expressed in reads per million.

Hierarchical clustering was performed on the 6000 most differentially-expressed transcripts across the HCT116 and CCD-18Co samples, and again across the HT29 and CCD-18Co samples using complete linkage clustering. From each cluster, transcripts were selected which showed enriched expression in CCD-18Co samples grown in 3D conditions, and non-redundant gene symbols for these transcripts were taken. Gene symbols in common between CCD-18Co cells grown in the presence of HT29 or HCT116 were chosen, and Ingenuity Pathways Analysis was performed using default analysis settings.

### *WNT* apoptosis and reporter assays

For apoptosis studies, CCD-18Co cells were infected with lentivirus expressing shRNAs to either *WNT2* or *WNT5A* or a non-targeting control shRNA, stained with a far-red tracking dye and mixed in a 50:1 ratio with HCT116-eGFP cells as previously described. After 72 hours of spheroid formation, spheroids were dissociated using non-enzymatic cell dissociation buffer (Gibco), stained with the Annexin-V/7-AAD Apoptosis kit (BD Biosciences) and subjected to flow cytometric analysis on a FACSCalibur (BD Biosciences). Cell quantitation was performed using FlowJo V10 software.

For WNT reporter studies, HCT116 cells were engineered to express a Super-Topflash (STF) luciferase reporter which is activated by the β-catenin-T-cell factor (TCF) transcriptional complex and is responsive to WNT signaling [52]. HCT116-STF cells were grown as monoculture spheroids or co-culture spheroids with CCD-18Co colon fibroblast cells. Spheroids were formed for 72 hours before treatment. Treatments included rhWnt5a protein (RnD Systems) at 25 nM or the porcupine inhibitor GNF-1331 at 100 nM. After 24 hours of treatment, spheroids were subjected to a Brite-Glo assay (Promega) according to the manufacturers’ instructions.

## SUPPLEMENTARY MATERIALS FIGURES AND TABLES








