# Supplementary material for: Functional profiling of microtumors to identify cancer associated fibroblast-derived drug targets
Source: Oncotarget. 2017 Oct 20;8(59):99913–30. doi: 10.18632/oncotarget.21915 (PMC5725140; doi:10.18632/oncotarget.21915)
Supplement: Supplementary file 1 [file oncotarget-08-99913-s001.pdf]

Functional profiling of microtumors to identify cancer associated fibroblast-derived drug targets

SUPPLEMENTARY MATERIALS

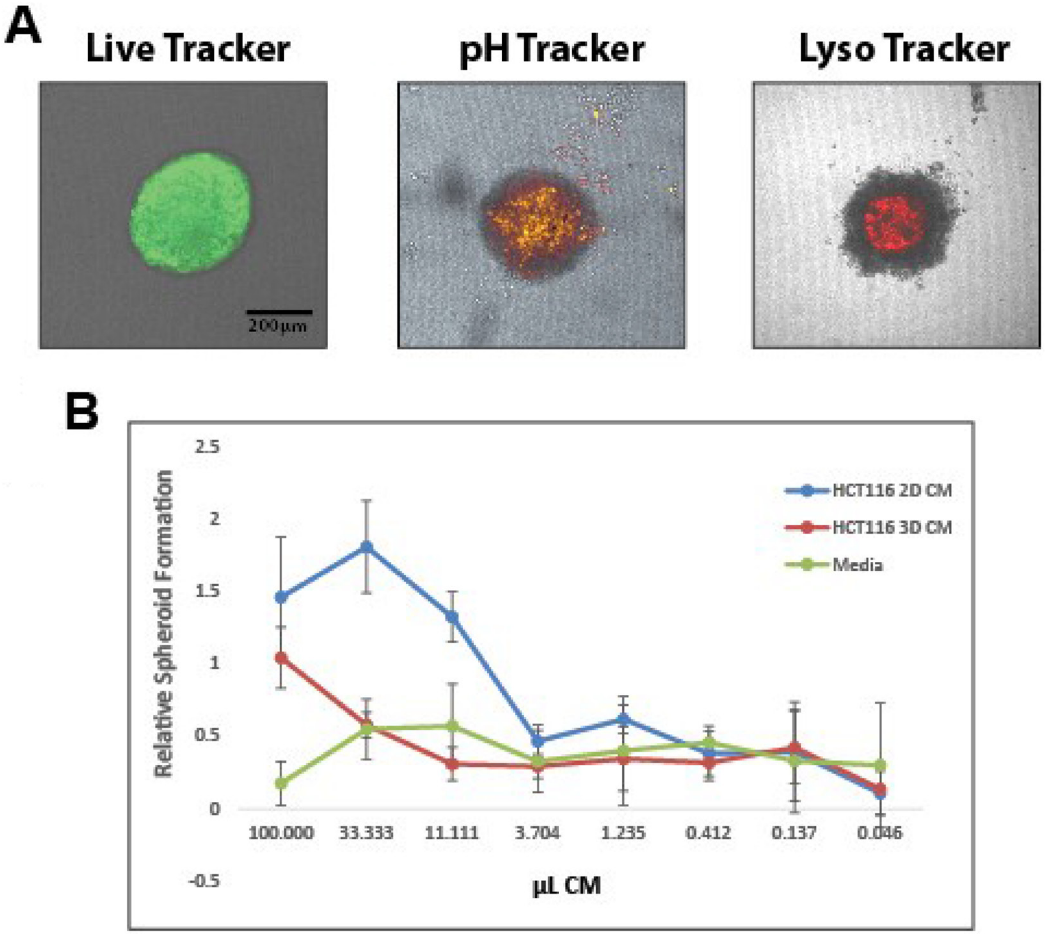

Supplementary Figure 1: Imaging analysis of acidic spheroid cores and effects of conditioned media on CAF monoculture spheroid formation. (**A**) Live tumor:fibroblast spheroids were stained with a live cell dye (Live Tracker), a pH-responsive dye (pH Tracker) or a lysosomal detection dye (Lyso Tracker) and imaged using laser confocal microscopy. Live, proliferating cells were mainly localized to the outer shell of the spheroid whereas the cores of spheroids stained strongly for low pH and lysosomal structures. (**B**) CCD-18Co cells grown in 3D hanging drops were able to be polarized into 3D spheroids by the addition of conditioned media (CM) from HCT116 cells grown as a 2D monolayer (blue) or as 3D spheroids (red) in a dose-dependent manner. Control media alone did not produce these 3D structures (green), indicated by < 1 value for spheroid formation.

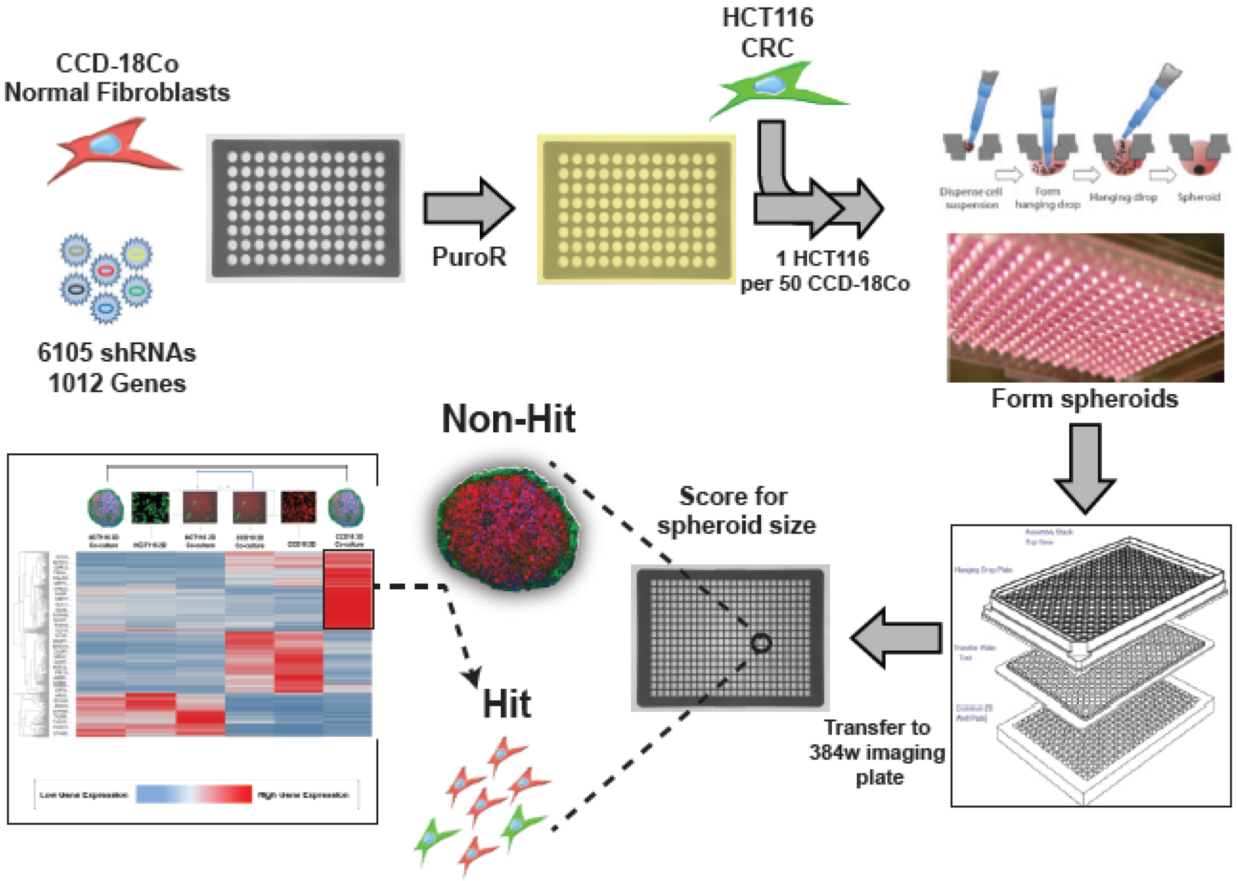

Supplementary Figure 2: Strategy for high-content tumor:stroma spheroid screen and hit triage. CCD-18Co normal human colon fibroblasts were transduced with 6,105 shRNA-expressing lentiviruses in arrayed fashion in 96-well plates. Positively-transduced cells were selected in puromycin for 72 hrs, stained with a far-red tracking dye then mixed in a 50:1 ratio with HCT116-eGFP human colorectal cancer cells. Cell mixtures were plated in 384-well hanging drops for 72 hrs to allow for spheroid formation. Spheroids were transferred to 384-well imaging assay plates using the 3D Biomatrix Spheroid Transfer Tool and spheroid formation was quantitated using laser-scanning fluorescence cytometry. Hit genes were characterized as having a strong anti-spheroid phenotype compared to non-hits. Further hit triaging was performed using RNAseq data from sorted co-culture spheroid models to identify genes that were upregulated more than 2.5-fold in 3D spheroid CAFs.

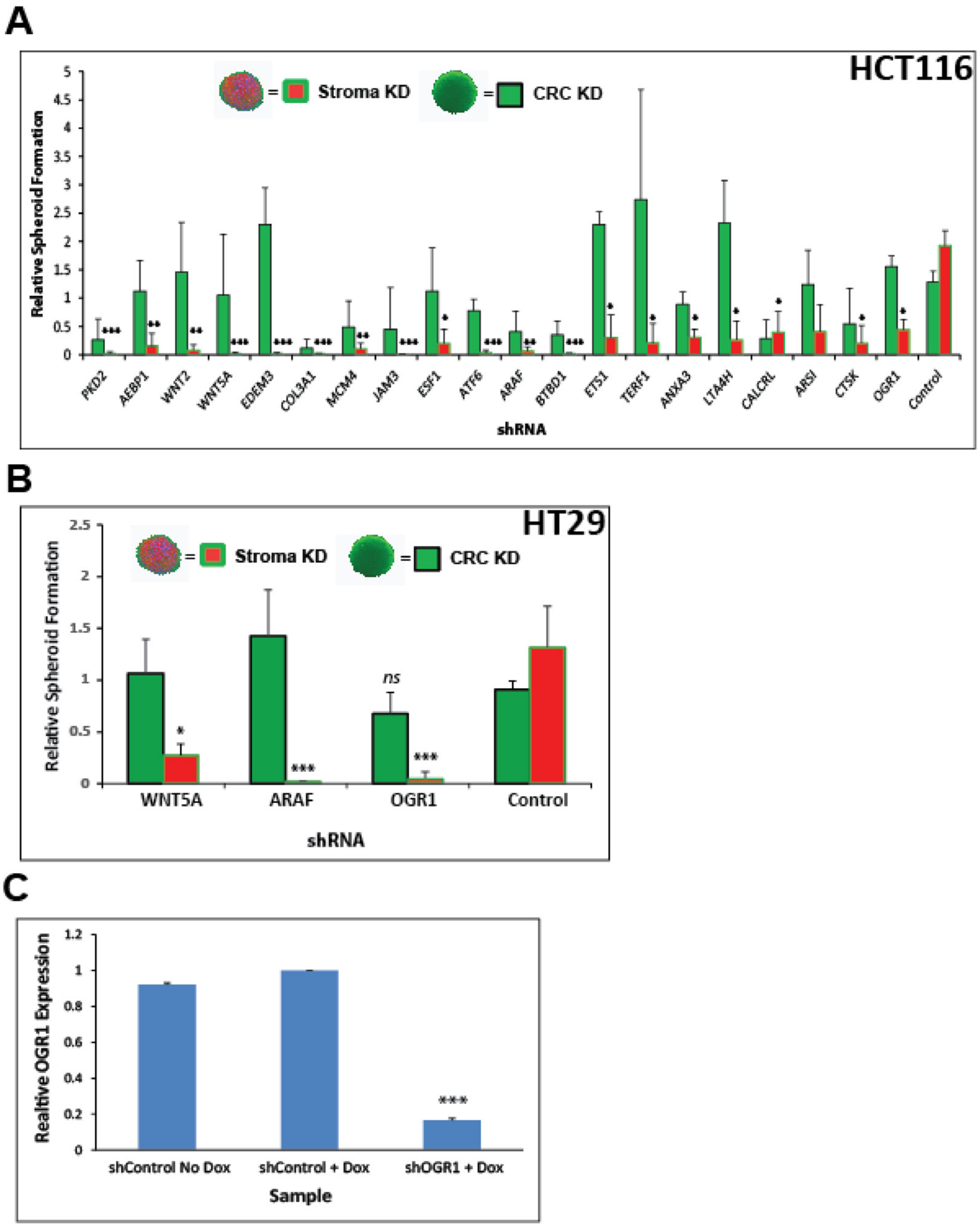

Supplementary Figure 3: Top 20 CAF-mediated targets identified from spheroid screen. (**A**) Top 20 targets from screen were knocked down using virally-mediated shRNAs either in HCT116 CRC cells in monoculture spheroids (green bars) or in CCD-18Co fibroblasts in co-culture spheroids (red/green bars). Most targets only exhibited an anti-spheroid phenotype when knocked down in the fibroblasts. (**B**) The same experiment as performed in Figure 6A, though with HT-29 human CRC cells. Knockdown of key target genes only had an effect on spheroid formation when occurring in the CCD-18Co fibroblasts in co-culture spheroids (red/green bars). (**C**) Quantitative PCR of *OGR1* under doxycycline-inducible knockdown of *OGR1* within MDA-MB-231 breast cancer cells (used for high *OGR1* expression) compared to a non-targeting shRNA control with and without doxycycline (1000 ng/mL) added. $^{*}p < 0.05$, $^{**}p < 0.01$, $^{***}p < 0.001$, *ns* = not significant.

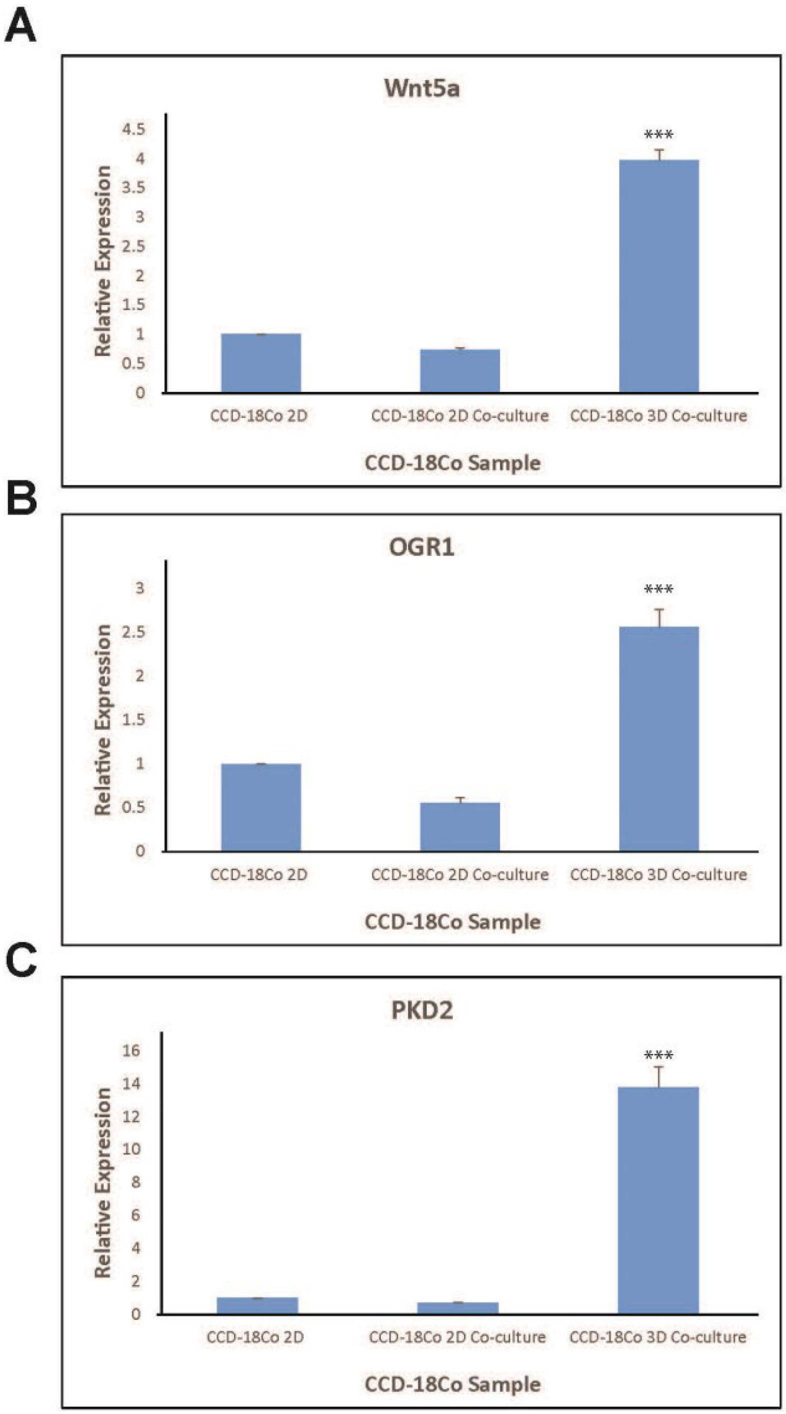

Supplementary Figure 4: Real-time PCR of Wnt5a, OGR1 and PKD2 from CCD-18Co cells grown either as 2D monoculture, 2D co-culture with HCT116 cells or 3D co-culture HCT116:CCD-18Co spheroids. $^{***}p < 0.001$.

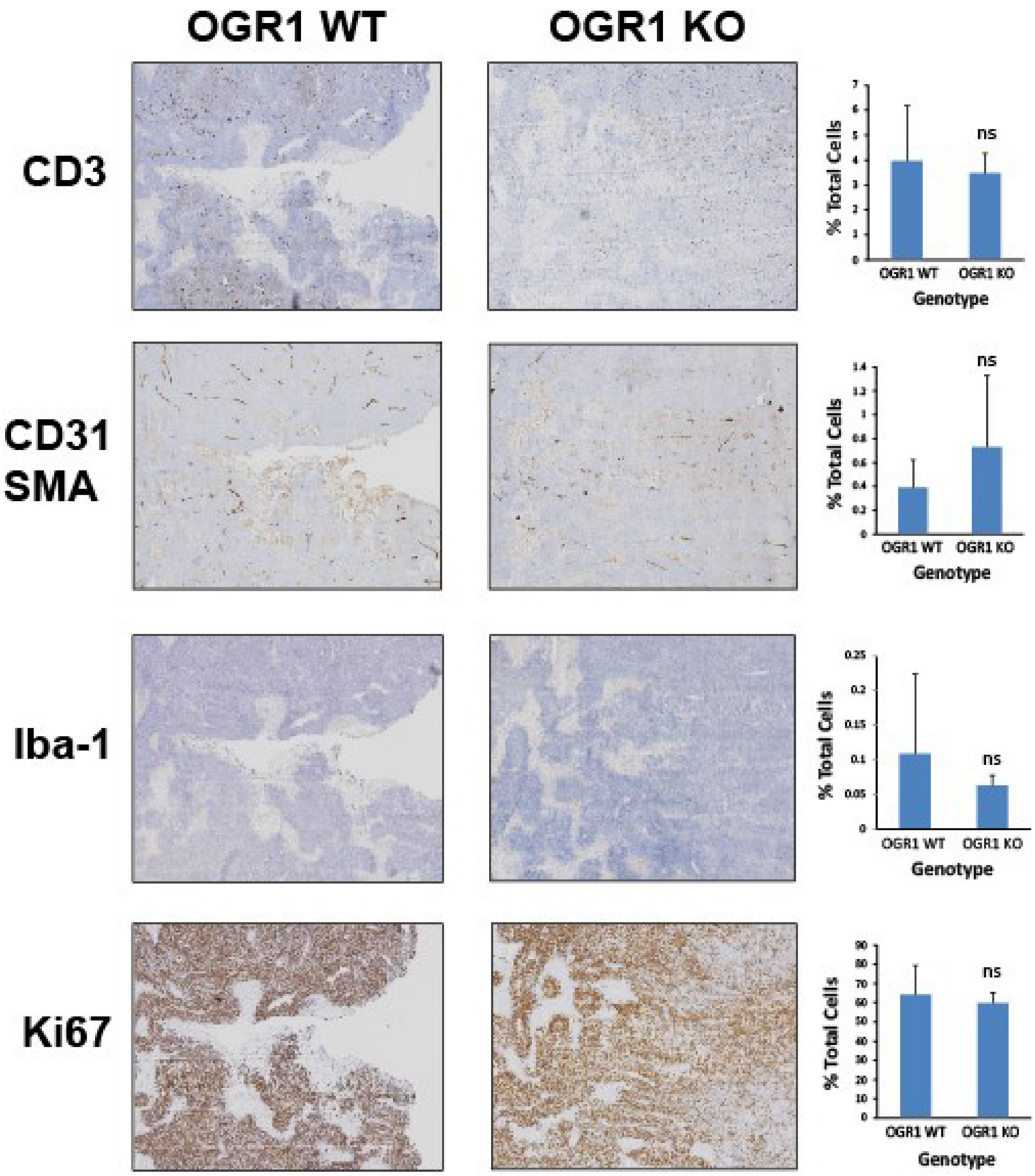

Supplementary Figure 5: Histological analysis of MC-38 tumors excised from Ogr1 WT or Ogr1 KO mice at 14 days post-implant. Histology images for T-cells (CD3), vasculature (CD31, punctate staining), fibroblasts (SMA, diffuse staining), macrophages (Iba-1) and cancer cells (Ki67) within MC-38 tumors. Inset graphs show field of view quantitations of respective cellular populations. ns = not significant.

Supplementary Table 1: List of 1,024 genes used in the high content tumor:stroma genomics screen as well as normalized screening data and scored values. See Supplementary_Table_1

Supplementary Table 2: RNAseq data from HCT116:CCD-18Co co-culture spheroids. See Supplementary_Table_2

Supplementary Table 3: RNAseq data from HCT116:CCD-18Co and HT-29:CCD-18Co co-culture spheroids. See Supplementary_Table_3

Supplementary Table 4: Results of OGR1 in situ hybridization on 95 CRC and 13 normal human colon tissue samples

Specimen	Positive Control Score	GPR68 Score	Histological description (Source)	Grade (source)	TNM Stage (T)	TNM Stage (N)	TNM Stage (M)
3554	2	2	Adenocarcinoma	G2	3	0	0
5037	2	2	Adenocarcinoma	G1	2	0	0
3322	2	1	Adenocarcinoma	G1	2	0	0
297456	2	1	Adenocarcinoma	G2	3	0	1
2926	3	1	Adenocarcinoma	G1	3	0	0
4400	2	2	Adenocarcinoma	G2	4	0	1
2585	2	1	Adenocarcinoma	G1	3	0	0
296507	3	3	Adenocarcinoma	G2	3	0	0
297840	3	2	Adenocarcinoma	G2	3	1	1
298436	2	1	Adenocarcinoma	G2	4	1	0
4177	2	3	Adenocarcinoma	G1	2	0	0
297273	3	1	Adenocarcinoma	G2	3	2	0
298181	3	2	Adenocarcinoma	G2	3	0	0
3026-69/10	3	1	Adenocarcinoma	G3	3	2	0
4005	3	4	Adenocarcinoma	G3	4	0	1
299579	3	1	Adenocarcinoma	G2	4	3	1
2706-29/10	3	4	Adenocarcinoma	G2	2	0	0
3918	3	1	Adenocarcinoma	G2	3	0	0
299265	3	1	Adenocarcinoma	G2	1	0	0
296816	3	2	Adenocarcinoma	G2	4	2	0
298963	3	1	Adenocarcinoma	G2	3	0	0
298156	3	1	Adenocarcinoma	G1	2	1	1
297523	3	1	Adenocarcinoma	G3	3	0	0
1905-35/10	3	1	Adenocarcinoma	G2	2	0	0
9615-33/10	2	1	Adenocarcinoma	G3	3	0	0
297522	3	2	Adenocarcinoma	G2	3	1	0

Histology Scoring; 1: Not stained, 2: Weakly stained, 3: Positively stained, 4: Strongly stained.
